# Silicon nanoparticles: A promising approach for control of *Pseudomonas aeruginosa* biofilms

**DOI:** 10.22038/IJBMS.2023.71088.15441

**Published:** 2023

**Authors:** Shaista Urooj, Zulfiqar Ali Mirani, Faraz Ahmed Pathan, Ghulam Mustafa, Mubashir Aziz, Bushra Jabeen, Sayed Hajan Shah, Asad Ullah, Najmul Hassan, Mohammad Naseem Khan, Yasir Raza Rajpoot

**Affiliations:** 1 Aquatic Diagnostic and Research Center Bahria University Karachi 75260 Pakistan; 2 Department of Microbiology, University of Karachi, Pakistan; 3 Microbiology Section, PCSIR Laboratories Complex Karachi-75280 Pakistan; 4 College of Food Science and Technology, Shanghai Ocean University, Shanghai 201306, China; 5 Centre for Development of Laboratory Equipment, PCSIR Laboratories Complex Karachi-75280 Pakistan; 6 Institute of Microbiology and Molecular Biology, Bahauddin Zakariya University, Multan, Pakistan; 7 Department of Prosthodontics, Dow International Dental Collage (DUHS) Karachi, Sindh-Pakistan; 8 Department of Biological Sciences, Government Degree Collage Larkana, Sindh-Pakistan; 9 Department of Physics and Oxide Research Center, Hankuk University of Foreign Studies, Yongin, 17035, Republic of Korea

**Keywords:** Biofilms, Nanoparticles, Pseudomonas aeruginosa, Silicon, SiNPs

## Abstract

**Objective(s):**

The current study aimed to investigate the control and treatment of biofilm-producing isolates of *Pseudomonas aeruginosa* using silicon nanoparticles (SiNPs).

**Materials and Methods:**

Biofilm-producing isolates of *P. aeruginosa* were recovered from various food samples and identified through fluorescent green colony formation on selective and differential media, as well as the amplification of *oprI *and *oprL* genes. Tube methods, Congo-red agar method, and scanning electron microscopy (SEM) were used to study biofilm phenotypes. The effect of SiNPs was evaluated by broth dilution assay.

**Results:**

The biofilm assay revealed that these isolates formed biofilms on glass surfaces within 72 hr of incubation. Scanning electron micrographs showed that the biofilm communities were composed of multicellular clusters of *P. aeruginosa* encased in matrix material. However, these isolates were unable to form biofilms on SiNPs-coated surfaces. The results showed that the planktonic isolates of *P. aeruginosa* were comparatively sensitive to the antibacterial properties of SiNPs, with minimum inhibitory concentration (MIC) ranging from 100 to 200 µg/ml. Contrarily, the biofilms were found to be 500 times more tolerant to the highest concentration of SiNPs (MIC of 500 µg/ml) and were more resistant. Under static conditions, the sedimentation of SiNPs resulted in their ineffectiveness. However, under shaking conditions, the biofilms were effectively dispersed and the cells were lysed. The results showed that SiNPs were effective against both the planktonic and the metabolically inactive forms of *P. aeruginosa.*

**Conclusion:**

This study suggests that SiNPs could be a useful tool for preventing the formation of biofilms and removing pre-existing biofilms.

## Introduction

Microbial food contamination is a significant issue that contributes to ongoing food crises worldwide (1). Microorganisms, including bacteria, fungi, and viruses, can cause deterioration of food quality, taste, and nutritional value (2). This contamination can happen due to inadequate handling, processing, packaging, and transportation (1, 2). Factors that promote the growth of microorganisms in food include inappropriate storage conditions, dirty equipment, contaminated raw materials, and poor hygiene practices by personnel (3). 

Moreover, improper maintenance of temperature, pH, and the use of preservatives can also create conditions that favor the growth of microorganisms in food (4). In addition, the inherent properties of food products, such as nutrient density, water activity, pH, food components, biological structures, humidity, and the presence of competing microorganisms, can also influence the growth of contaminants (5). Microorganisms are capable of producing a wide range of spoilage metabolites, such as alcohols, sulfur compounds, hydrocarbons, fluorescent pigments, organic acids, esters, and carbonyls, that can cause food to become unpalatable (6, 7). The consequences of microbial food contamination include food-borne illnesses, food waste, and economic loss. Diarrhea, dysentery, and typhoid are some of the well-known food-borne infections (8). Research suggests that every year, one-third of the global population is affected by food-borne infections caused by microorganisms (9). The most frequently recognized food-borne infections are caused by bacterial pathogens such as *Escherichia coli*, *Salmonella*, *Staphylococcus* *aureus*, and *Pseudomonas* *aeruginosa* (10, 11). These pathogens are capable of growing and surviving in a wide range of conditions (9, 10). They can be transmitted into food through adhesion to equipment surfaces and the hands of food handlers (12). Additionally, many pathogens can form biofilms, which provide a means of survival under adverse conditions. Previous studies have shown that biofilms are one of the most resistant forms of bacterial existence (13, 14). Bacteria have the ability to adhere to solid surfaces and protect themselves by producing extracellular matrix materials (14). This makes it difficult for a variety of antibacterial agents to penetrate the matrix, rendering them ineffective (15). Bacteria in biofilm communities can persist and persistently contaminate food products for extended periods of time (13-15). This makes bacteria that produce biofilms a significant challenge for the food industry (16).

 One of the biggest challenges in food science is controlling and reducing contamination in food products (12). There are various strategies used to achieve this goal and improve food product quality (17). Among these, nanoparticles are the most effective agents for disinfecting various food items (18). Nanoparticles with sizes ranging from 1 to 100 nanometers have been shown to be highly effective in both aqueous and solid media (19). Silver (Ag) and gold (Au) based nanoparticles are widely used for decontamination in the pharmaceutical and food industries due to their antimicrobial properties and low toxicity to mammalian cells (20). Silica nanoparticles (SiNPs) are also being used in food preservation and packaging (21). Previous studies have demonstrated that silicon or silica is widely used in industrial and biomedical applications (22). Recent research has shown that SiNPs are a practical option due to their modifiable surface, desirable mechanical properties, and relatively non-reactive chemical composition (23). Silica NPs are considered a powerful group of nano-carriers for delivering anti-microbial drugs, due to their biocompatibility and porous surface (24). These NPs may also be an effective solution for controlling multi-species biofilms, they concurrently target and combat numerous drug-resistant pathogens. (24, 25). Additionally, SiNPs have the advantage of possessing a high level of roughness and penetration ability, enabling them to effectively penetrate the biofilm and eradicate pathogens more effectively than other NPs with lower roughness (24, 25). They are equally effective against both vegetative and dormant bacterial cells, bacterial fragments, viruses, and fungi (25). SiNPs are also used for decontaminating the surfaces of packaging equipment (24, 25). Given these benefits, this study was designed to assess the efficacy of SiNPs against biofilms of *P. aeruginosa*. 

## Materials and Methods

***Isolation and confirmation of bacterial strains***

The current study aimed to assess the effect of SiNPs on the biofilm of *P. aeruginosa*. The subject isolates were obtained from different samples of spices mix and sweet mix. The isolated strains were grown on Pseudomonas Cetrimide agar (OXOID, UK) and Pseudomonas Agar Base with CN selective supplement (OXOID, UK). After incubation of 24 hr at 35 °C, fluorescent green colonies appeared. Afterward, a polymerase chain reaction (PCR) was run for confirmation. Pseudomonas was confirmed by using primers against the* oprI *gene*. *For the confirmation of *P. aeruginosa oprL *was used (26). The sequences of primers used in the study are *oprI*-F 5-’ATGAACAACGTTCTGAAATTCTCTGCT-3’, *oprI*-R 5’CTTGCGGCTGGCTTTTTCCAG3’, *oprL-*F 5-’ATGGAAATGCTGAAATTCGGC-3’, *oprL-*R-5’CTTCTT 

CAGCTCGACGCGACG3’. The experimental conditions included an initial activation at 94 °C for 5 min, followed by denaturation at 94 °C for 1 min, annealing at 55 °C for 1 min, elongation at 72 °C for 1 min, and a final extension at 72 °C for 10 min.

***Preparation of SiNPs***

The synthesis of silicon nanoparticles (SiNPs) was conducted based on the method developed by Zulfiqar *et* *al*. (27). The procedure involved preparing a mixture of 33% ammonia and ethanol in a 1:1 ratio (30 ml each). A precursor medium was then created by adding 0.5 ml of 25% sodium silicate to 7 ml of deionized water. This precursor solution was added dropwise to the mixture of ammonia and ethanol. Characterization of the SiNPs revealed an average particle size of 94±30nm. Energy-Dispersive X-ray Spectroscopy (EDX) was employed for additional characterization of the synthesized SiNPs. EDX analysis confirmed the presence of silicon as the primary element in the SiNPs, validating their identity. The technique also provided insights into the purity of the nanoparticles by detecting the absence of significant impurities or foreign elements.

***Antimicrobial activity of SiNPs***

The antimicrobial activity of SiNPs against subject isolates of *P. aeruginosa* was performed by the standard broth dilution method [CLSI M07-A8] (28). A serial two-fold dilution of SiNPs in concentrations ranging from 5 µg/ml to 1000 µg/ml was adjusted in tryptone soya broth (TSB). The overnight culture of *P. aeruginosa* was adjusted to 10^6^ CFU/ml and 0.1 ml inoculated in TSB followed by incubation at 35 °C. After 24 hr of incubation, the growth was monitored and the MIC of SiNPs was noted. 

***Biofilm assay***

The biofilm formation was examined according to the standard tube method. Briefly, overnight cultures (0.1 ml) of *P. aeruginosa* were inoculated into 100 ml TSB flasks (pH=7.0) and incubated at 35 °C for 24, 48, 72, and 96 hr. Sterile rectangular glass slides measuring approximately 25 mm x 75 mm with smooth, flat surfaces were then placed in each flask with the lower half submerged in broth. After incubation, these slides were collected and washed twice with phosphate buffer saline and stained with crystal violet as described earlier by O’Toole [24]. The cell-bound crystal violet was dissolved in 33% acetic acid, and biofilm thickness was measured in terms of optical density (OD) at 570 nm. The two glass slides in TSB without *P. aeruginosa *were used as negative control (29).

***Exposure of biofilms to SiNPs***

The sterilization process for the SiNPs involved exposure to ultraviolet light. One milligram per milliliter of the SiNPs was placed in a sterile glass tube and exposed to UV radiation for 3 hr to ensure sterility. In order to assess the impact of SiNPs on biofilm formation, glass slides were used as a substrate, as previously mentioned. The biofilm was allowed to establish with a density of approximately OD 0.97 up to 96 hr and exposed to SiNPs for 48 hr at concentrations ranging from 100 µg/ml to 1000 µg/ml. The slides were examined at multiple intervals, starting with the first observation at 0 hr and concluding with the final observation after 48 hr. After the incubation period, the slides were gently removed and washed three times with 0.2 ml of phosphate buffer saline (PBS, pH 7.2) to eliminate debris and unbound cells. The slides were then placed back into fresh TSB flasks and incubated for an additional 24 hr at 35 °C, during which the growth was monitored. The absence of turbidity in the TSB flask, in the presence of the corresponding dose of SiNPs, was considered as the MIC. This indicated that the concentration of SiNPs used was sufficient to inhibit the growth of the biofilms. For the quantification of viable cells, a slide was collected from the flask and immersed in 1 ml of phosphate buffer saline (PBS). The slide was then subjected to vortexing at 500 rpm for 1 min to dislodge the cells from the slide surface. From this suspension, 1 ml was carefully poured onto a plate containing Tryptone Soy Agar (TSA) medium. The TSA plates were subsequently incubated for 24–48 hr at 35 °C, allowing the viable cells to grow and form visible colonies. After the incubation period, the colonies were counted to determine the number of viable cells present on the slide.

Additionally, to simulate different environmental conditions, the biofilm-exposed slides were incubated both in a static environment and in a shaking incubator set at 200 RPM. To monitor the progression of biofilm formation and SiNPs’ effects over time, the slides were collected at specific intervals: 30 min, 1 hr, 1.5 hr, 2 hr, 3 hr, 4 hr, 6 hr, 12 hr, 18 hr, 24 hr, 36 hr, and 48 hr. Before any measurements were taken, the slides were carefully washed with distilled water to remove any potential interference. To visualize and quantify the thickness of the biofilm, the stained crystal violet method was employed. After washing, the stained biofilm on the glass slides was examined, and the thickness of the biofilm was determined by measuring the optical density at 570 nm, offering insights into the growth and development of the biofilm under the influence of SiNPs.

***Effect of SiNPs; scanning electron microscopy (SEM) and energy-dispersive x-ray spectroscopy (EDX)***

SEM in conjunction with EDX was employed to conduct a comprehensive examination of the biofilm’s structure and to definitively verify the elemental composition of the SiNPs. Biofilm-positive slides were divided into 4 mm sections, washed with distilled water to remove debris, and negatively stained with 2% uranyl acetate for 30 sec. Dehydration was carried out with absolute ethanol, initially at 50% for 30 min, followed by 75% for 30 min, and 95% for 30 min. The samples were then coated with platinum by the auto-fine coater (JEC-3000FC) at a 20-mA current in a vacuum for 30 sec. Images were obtained using a JSM IT 100 JEOL. The electron microscope utilized a tungsten electron beam source under high vacuum conditions for high-resolution imaging, capturing images at varying electron voltages (5 to 20 KV) depending on the sample, with a working distance of 5–10 mm from the pole piece, and acquiring images using a Secondary Electron detector (SEI). The sterilized nanoparticles were carefully mounted on SEM stubs using adhesive tape and uniformly coated with carbon (JEOL-EC-32010CC). The samples were placed in the sample chamber of the SEM-EDS (JEOL JSM-IT 100, Japan) and scanned under different magnifications, ranging from ×6000 to ×8000, at a voltage of 20 kV, as previously recommended (29).

***Biofilm formation on SiNPs coated glass slides***

The glass slides were cleaned with acetone for 10 min with ultra-sonication and dried. The SiNPs (5 mg/ml) were deposited onto the glass slides by spray coating and then the film was desiccated under oven heat at 150 °C for 1 hr. The intensity of SiNPs film and surface characterization was confirmed by SEM. These slides were placed in 100 ml TSB broth and inoculated with biofilm-producer *P. aeruginosa*. These tubes were incubated at 35 °C for 96 hr. The plain glass slides were used as the negative control. The OD of biofilms was recorded after 24, 48, 72, and 96 hr. The slides were washed with de-ionized distilled water and stained with crystal violet. The biofilm thickness was measured in terms of OD at 570 nm (29).

## Results

A total of ten *P. aeruginosa* isolates were selected for this study based on their ability to form biofilms. These isolates were obtained from various food samples and identified using characteristic fluorescent green colonies on Pseudomonas Cetrimide agar and Pseudomonas Agar Base with CN selective supplement (both from OXOID, UK). Confirmation of the isolates was performed using an oxidase test and amplification of the *oprI* and *oprL* genes through PCR (Supplement 1).

***Characteristics and antimicrobial activity of SiNPs***

**Figure 1:**
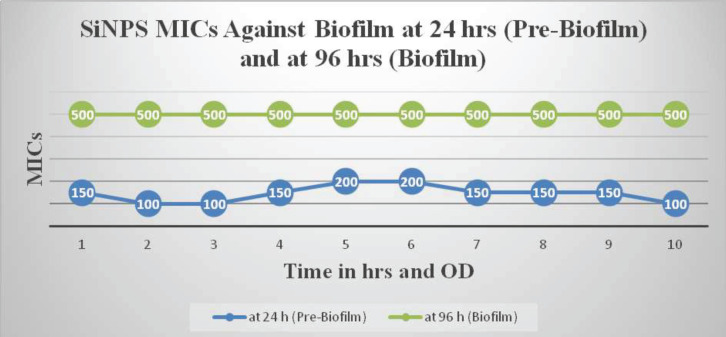
Comparison of MIC of SiNPs against pre-biofilm and biofilm stages of *Pseudomonas aeruginosa*

EDX and SEM were employed to characterize the SiNPs. The analysis revealed that the SiNPs had an average particle size of 94±30 nm. They exhibited a spherical shape and a smooth surface. The particles showed minimal size variation, with a range of ±30 nm. The antibacterial assay results showed that *P. aeruginosa* in its planktonic form was susceptible to SiNPs, with a MIC ranging from 100 to 200 µg/ml for most isolates (Figure 1). However, biofilm formations were highly resistant, displaying a 5-fold increase in tolerance with a MIC of 500 µg/ml.

**Figure 2:**
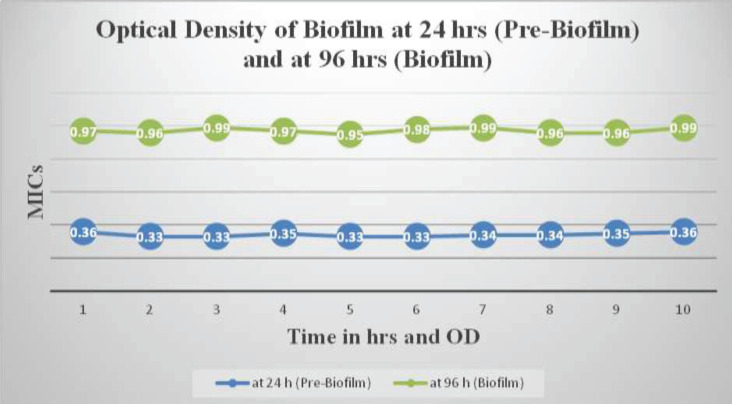
MIC of *Pseudomonas aeruginosa* biofilm formation over time

The data demonstrate that mature biofilms of *P*. *aeruginosa* exhibit a higher level of resistance compared to their planktonic counterparts. The MIC values for the planktonic stage were 100 µg/ml, 150 µg/ml, and 200 µg/ml. However, after adopting the biofilm lifestyle, the isolates showed increased resistance to SiNPs, with MIC values of 500 µg/ml (Figures 1 & 2).

***Biofilm formation***

*P. aeruginosa* was allowed to form biofilm on a glass surface and after 96 hr of incubation at 35 °C, a solid and substantial biofilm consortium was observed with an optical density of 0.97 (Figure 2). Scanning electron micrographs further confirmed the formation of a multi-layered structure of the biofilm consortium, covered with an extracellular matrix (Figure 5). The isolates selected for this study were based on the intensity of their biofilm formation, with optical densities less than 0.95.

***Effect of SiNPs on multi-layered biofilms***

**Figure 3:**
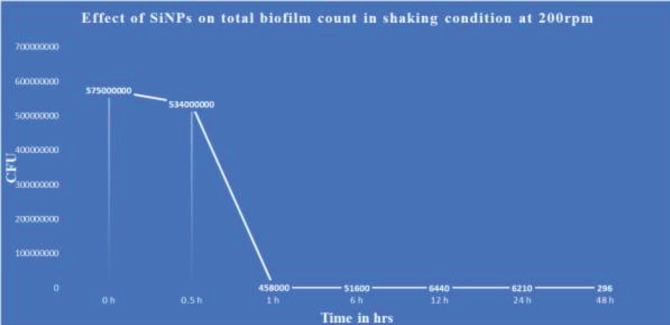
The graph shows the effect of SiNPs (500 µg/ml) on *Pseudomonas aeruginosa* population/CFU with exposure time and shaking at 200 rpm

The mature biofilm consortia were developed after 96 hr of incubation with an average optical density (OD) of 0.97. The biofilm consortia were highly organized and strongly adhered to the surface, making them challenging to disperse. This was confirmed through the application of a vortexer and sonicator. The biofilm-positive slides were vortexed at a speed of 500 RPM for 2 min and sonicated for 30 min using a 40 kHz sonicator. A minor reduction in biofilm density was observed, with the average initial OD of 0.97 decreasing to 0.91 and 0.87 after vortexing and sonication, respectively. However, exposure to 500 µg/ml SiNPs resulted in significant dispersion of these highly organized and strong biofilm consortia of *P. aeruginosa*. The slides were placed in 100 ml PBS with 500 µg/ml SiNPs and incubated at 35 °C. One flask was placed in a static position, while the other was placed in a shaker at 200 RPM. Initially, the established biofilm consortia were exposed to 500 µg/ml of SiNPs for 30 sec. However, no change in biofilm intensity was noticed at this stage (Figure 3). The gradual increase in exposure time with 500 µg/ml of SiNPs resulted in the gradual destruction of biofilm consortia (Figure 3). The biofilm OD was recorded after 30 min, 1 hr, 1.5 hr, 2 hr, 3 hr, 4 hr, 6 hr, 12 hr, 18 hr, 24 hr, and 48 hr (Figure 3). The results showed a gradual reduction in biofilm OD. After 1 hr, a 29% reduction in biofilm OD was recorded. A 1.5-hour exposure to SiNPs resulted in a 30% dispersion of the consortia. The biofilm was wiped out by 58% in 4 hr and 79% in 12 hr with 500 µg/ml of SiNPs. After 24 hr of exposure to SiNPs, 87% of the biofilm consortia were dispersed from the surface of the glass slides. More than 90% of the biofilms were removed after 48 hr of treatment with SiNPs under shaking conditions at 200 rpm (Figure 3). 

**Figure 4:**
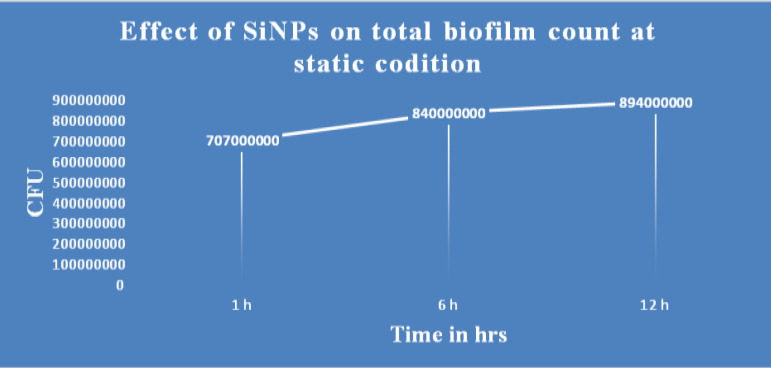
Effect of SiNPs (500 µg/ml) on the biofilm population of *Pseudomonas aeruginosa* in static conditions is shown in this figure

**Figure 5:**
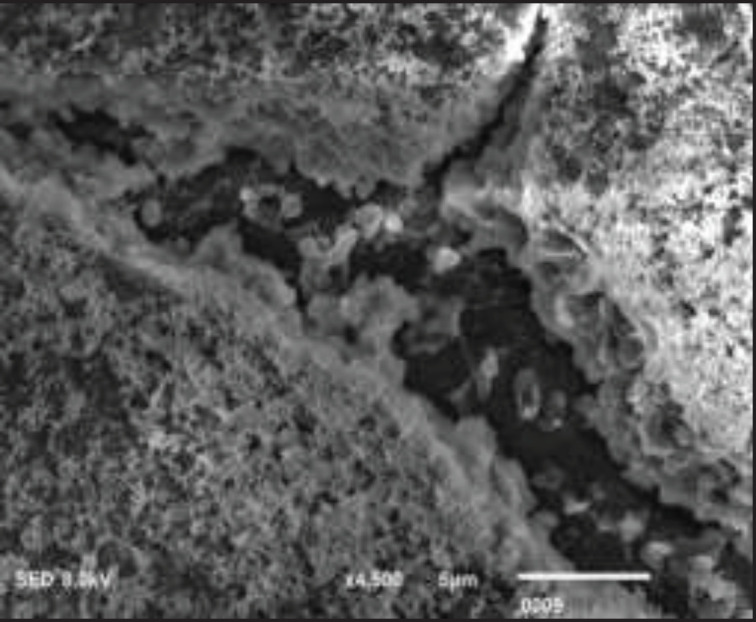
Scanning electron micrograph of *Pseudomonas aeruginosa *biofilm on glass slides

In comparison, the slides placed in a static position were comparatively resistant to the effects of SiNPs and showed a slower but steady increase in the density of the biofilm consortia (Figure 4). For example, the average OD of the biofilm consortia was 0.97 at the initial exposure time, 0.99 after 12 hr, and 1.01 after 24 hr. Further analysis of the buffer indicated that the SiNPs settled at the bottom of the flask within a minute without disturbing the bacteria attached to the slide surface in the biofilm consortia (Figure 5). The population analysis assay revealed a similar trend in cell count. Initially, the count of *P.* *aeruginosa* cells in the biofilm consortia was 5.7x10^8^ colony-forming units CFU/cm^2^, and no significant reduction was observed after 30 min of exposure to 500 µg/ml SiNPs under shaking conditions. However, a noticeable decrease was seen after 1 hr of exposure (Figure 3).

***Biofilm formation on SiNPs coated slides***

**Figure 6:**
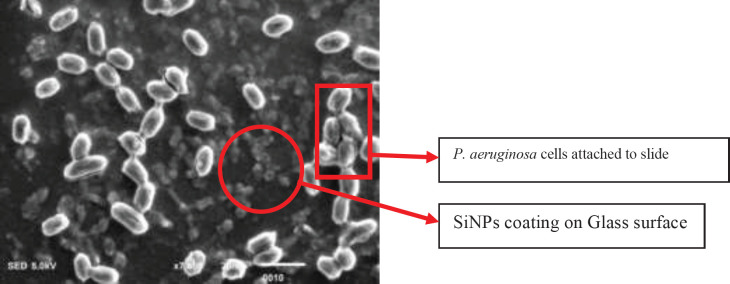
Scanning electron micrograph of biofilm on SiNPs-coated slides

The SiNPs coated slides were used to provide a solid base for biofilm formation. The comparative analysis revealed that all the subject isolates of *P. aeruginosa* effectively produced biofilm. However, these isolates were unable to adopt the biofilm lifestyle on SiNPs coated slides. The mean OD of biofilms for all 10 isolates on the glass slide was 0.97 (Figure 6). On the contrary, the mean OD on SiNPs coated slides was 0.24 after 96 hr of incubation. Furthermore, the spot assay indicated a zone of inhibition around these SiNPs coated slides (Supplement 2).

***Penetration of SiNPs inside the biofilm consortia***

**Figure 7:**
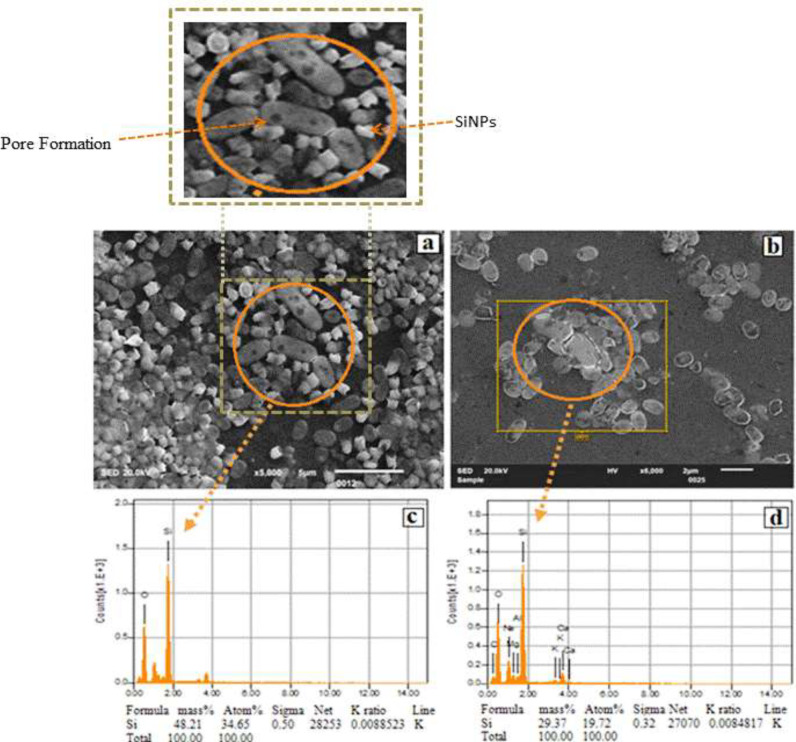
(a & b) Scanning electron micrographs showing the presence of SiNPs surrounding *Pseudomonas aeruginosa* cells, and the formation of micro-pores in nearby cells. (c &d) The EDS spectra are confirming the presence of SiNPs

SiNPs: Silicon nanoparticles; EDS: Energy-dispersive X-ray spectroscopy

**Figure 8:**
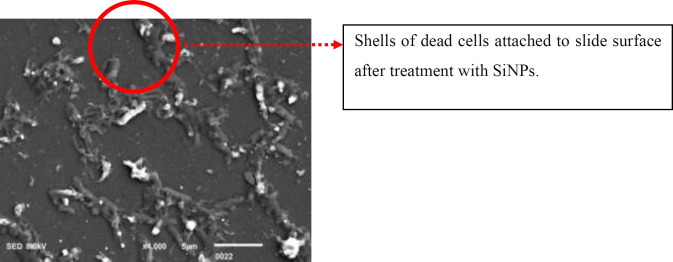
SEM image of dead cells attached to the surface after exposure to SiNPs

The SiNPs were not able to penetrate the consortia under static conditions. As a result, the biofilm OD increased even in the presence of 500 µg/ml of SiNPs. Conversely, in shaking conditions at 200 rpm, the SiNPs were able to cross the extracellular matrix material and penetrate the consortia. The cells on the surface and in the middle of the consortia were lysed and dispersed, and the population analysis assay showed that most of the cells were unable to survive. The cells at the lower base of the consortia were strongly attached to the glass surface. Scanning electron micrographs revealed that SiNPs exterminated these cells by creating a stoma in the cell wall. The scanning electron micrographs also indicated that SiNPs adhered to the cells’ surface and ruptured the cell wall (Figure 7 a & b). The results demonstrated that shaking at 200 rpm facilitated the penetration of SiNPs into the matrix, leading to cell lysis within the consortia. Scanning electron micrographs revealed that remnants of deceased cells remained firmly attached to the surfaces (Figure 8). 

## Discussion

Biofilm is a highly resistant state of bacterial life that is often adopted by bacteria in response to harsh environments (30, 31). It is a survival strategy that enables bacteria to nullify the toxic effects of antibacterial agents, predators, invaders, and starvation (32). Biofilms are widely prevalent in clinical settings and food processing units and are found wherever bacteria exist. Despite efforts to control biofilms using different antibacterial agents such as AgNPs, SeNPs, ascorbic acid, and fatty acids, they are still a persistent problem (33). In this study, a new approach was taken to control biofilms of *P. aeruginosa* using SiNPs. Results showed that SiNPs were effective against both planktonic and biofilm phenotypes of *P. aeruginosa*. The planktonic population was sensitive to low concentrations of SiNPs, but the biofilm population was resistant and able to survive even at the highest concentration of SiNPs. The effectiveness of SiNPs against the biofilm of *P. aeruginosa* is supported by the findings of Smirnov *et* *al.* (34). The scanning electron micrographs revealed that these particles adhered to the surface of bacteria and created pores in the cell walls (Figure 7). The SiNPs were effective against both the vegetative and metabolically inactive cells, with the vegetative cells being more sensitive. However, the metabolically inactive or slow-growing population, found mostly at the base of the biofilm consortia, was more resistant and persisted for a longer time (14, 29). Previous research has shown that biofilm consortia can harbor multiple phenotypes including planktonic, slow-growing, and metabolically inactive or persister cells, and conventional drugs are often unable to control or disperse these phenotypes (14). However, the SiNPs effectively reached the lower base of the biofilm consortia and penetrated the persister and metabolically inactive cells of *P. aeruginosa* (Figure 6). The use of SiNPs as antibacterial agents has a unique advantage over traditional drugs. Many drugs are unable to penetrate the protective layer of extracellular matrix material that surrounds biofilms, making them ineffective against the bacterial populations within (34, 35). However, the SiNPs were found to be able to cross this barrier and effectively target all phenotypes within the biofilm consortia, including the pre-existing biofilms of *P. aeruginosa* in this study (Figures 6 & 7).

Initially, the effectiveness of the SiNPs was limited by their tendency to sediment in static conditions. To overcome this, the pre-existing biofilm slides were exposed to the SiNPs in a shaking incubator set at 200 rpm, which prevented sedimentation and kept the particles in a floating state. The SiNPs were able to penetrate the biofilms and disperse the consortia. They not only penetrated but remained within the consortia, targeting and dispersing the pre-existing biofilms in a short period of time. Previous studies have shown that metabolically inactive and slow-growing phenotypes within biofilm consortia are more hydrophobic (14, 36, 37). Despite this, the SiNPs were still effective against these populations, demonstrating their potential as a valuable tool in controlling biofilms. Therefore, these metabolically inactive and slow-growing phenotypes are highly adhesive and persist for a long time in a dormant state. According to a study by Wang *et* *al*. (11), hydrophobic particles easily penetrate bacteria, while hydrophilic particles are trapped outside the extracellular matrix materials. The SiNPs exhibited high stability in aqueous media (28) and effectively targeted metabolically inactive, slow-growing, and planktonic *P. aeruginosa*. Upon adherence, SiNPs created pores in the cell wall and membrane, leading to cytoplasmic content leakage and cell lysis (Figure 7).

A study (38) suggested that the SiNPs adhered to the surface of bacteria by forming hydrogen bonds with amino acid residues. Smirnov *et* *al*. (34) also suggested that the presence of single oxygen free radicals and other reactive oxygen species on the surface of the SiNPs leads to oxidative damage to bacterial membranes and ultimately death of the bacterial cell. This is considered a possible and probable mechanism of antibacterial action. An interesting observation from this study was the strong attachment of *P. aeruginosa* cells to glass slides, even after cell death (Figure 8). Our studies on biofilm suggest that the cells at the base of the consortia are holding the burden of the consortia (39, 40). Therefore, these phenotypes strongly adhere to the surface and carry the weight of the consortia (39, 40). They are mostly dormant or metabolically inactive due to starvation, suffocation, and congestion at the lower part of the consortia (14, 39). This study found that these phenotypes maintain their adhesion even after the death of the consortia (Figure 8). The majority of antibacterial agents are unable to target these phenotypes (41). However, the SiNPs were found to effectively penetrate the extracellular matrix material and disrupt the power-bearing phenotypes of the consortia (Figures 6 & 7). This is also supported by other studies, which showed that the lower base of biofilms is occupied by dead, compromised cells (40, 42). The heterogeneous environment and population of biofilms make them highly resistant to most antibacterial agents (39, 40). In addition, the glass slides coated with SiNPs were found to reduce the adhesion of *P. aeruginosa* to the surface (Figure 7). This makes it a promising option for increasing the shelf life of food and enhancing the effectiveness of packaging materials. Silicon dioxide nano-materials have been authorized for use in food packaging in Europe, as silicon has been safely used in food packaging for a long time (43, 44). Moreover, these particles are non-leaching and stable on the surface for a prolonged period (43). This has been validated by the studies of Akhidime *et* *al*. (45). The findings of the present study, as well as those of previous researchers, have shown that SiNPs effectively eliminate bacteria from the media. Biofilms are known to be highly resistant states of bacterial life, making this a significant achievement. In a remarkable achievement, SiNPs successfully eradicated the existing *P. aeruginosa* biofilm within 48 hr, which is known for its resilience. This study highlighted the efficacy of SiNPs against both free particles and coating material, emphasizing the need for additional *in vitro* and *in vivo* investigations to unlock their potential for food preservation.

This figure illustrates the comparison of SiNPs' MIC against both pre-biofilm and biofilm stages of *P. aeruginosa. *The MIC for biofilms was determined to be 500 µg/ml, while the pre-biofilm MIC ranged between 150 µg/ml and 200 µg/ml.

MIC: Minimum inhibitory concentration; SiNPs: Silicon nanoparticles

The graph illustrates the progression of the biofilm thickness of *P. aeruginosa* over time. After 96 hr of incubation, the biofilm transitions from an early, weak stage to a mature, dense state. The OD measurement of the biofilm (sessile cells) was recorded after 96 hr of incubation, exceeding 95. In contrast, at 24 hr, the biofilms were significantly weaker with an OD measurement of less than 36.

MIC: Minimum inhibitory concentration; OD: Optical density

The biofilm was densely populated, but after exposure to SiNPs, a gradual decrease in cell numbers was observed, with most cells being eliminated after 48 hr of incubation. The initial CFU count was measured at 5.7x108. Following exposure to a concentration of 500 µg/ml, the CFU count exhibited a gradual reduction over a 48-hr period, reaching a final count of 2.9x10^2^.

SiNPs: Silicon nanoparticles; CFU: Colony forming units

The results indicate that SiNPs were not effective in these conditions and the cell population gradually increased over time. The initial CFU count was recorded as 7.0×10^8^. After a 12-hr exposure to 500 µg/ml SiNPs under static conditions, the CFU count slightly increased to 8.9×10^8^.

SiNPs: Silicon nanoparticles; CFU: Colony forming units

The images show cellular aggregates of the biofilm, which are held together by extracellular matrix material. The thickness and structure of the biofilm can be observed through these SEM images.

The images show the absence of cellular aggregates and extracellular matrix material on the SiNPs-coated surfaces after 96 hr of incubation with *Pseudomonas aeruginosa.*

SiNPs: Silicon nanoparticles

The cells, which were located at the lower base of the biofilm consortia, have died but remain adhered to the surface

SEM: Scanning Electron Microscopy; SiNPs: Silicon nanoparticles

## Conclusion

The findings of the current study indicate two key benefits of using silicon dioxide nanoparticles (SiNPs). Firstly, they were able to effectively disperse pre-existing biofilms of *P. aeruginosa*. Secondly, coating surfaces with SiNPs prevented bacterial adhesion and biofilm formation. The stability and efficacy of SiNPs were demonstrated against both planktonic and biofilm phenotypes of *P. aeruginosa*. These particles were able to penetrate the complex structure of the biofilm consortia and target various phenotypes, including planktonic, slow-growing, and dormant cells. By binding to the cell surface and creating pores in the cell wall and membrane, SiNPs likely killed the target cells. Based on these results, SiNPs show potential as a suitable candidate for use in industrial food preservation and packaging materials. Further *in vitro* and *in vivo* studies are recommended to further examine the role of these highly effective compounds.

## Authors’ Contributions

ZA M designed and supervised the study. FA P and S U contributed to experiment and write-up. G M prepared the SiNPs. S HS and MN K contributed to experiment and write-up. Asadullah helped with characterization of SiNPs. N H and YR R reviewed the study design. M A and B J helped with revision, English language, and grammatical correction.

## Conflicts of Interest

The authors declare that no conflict of interest exists.

## References

[1] Cheng H, Xu H, McClements DJ, Chen L, Jiao A, Tian Y (2022). Recent advances in intelligent food packaging materials: Principles, preparation and applications. Food Chem.

[2] Maraz KM, Khan RA (2021). An overview on impact and application of microorganisms on human health, medicine and environment. GSC Biol Pharm Sci.

[3] Mutua A (2021). Role of food management systems on food safety in hotels. J Food Sci.

[4] Vishweshwaraiah YL, Acharya A, Hegde V, Prakash B (2021). Rational design of hyperstable antibacterial peptides for food preservation. NPJ Sci Food.

[5] Hallsworth JE (2022). Water is a preservative of microbes. Microb Biotechnol.

[6] Banerjee DK, Das AK, Thakur N, Talukder S, Das A, Sonowal J (2019). Factors affecting microbial growth in livestock products: A review. IJCS.

[7] Díaz-Montes E, Castro-Muñoz R (2021). Edible films and coatings as food-quality preservers: An overview. Foods.

[8] Aworh OC (2021). Food safety issues in fresh produce supply chain with particular reference to sub-Saharan Africa. Food Control.

[9] Fouladkhah AC, Thompson B, Camp JS (2019). Safety of food and water supplies in the landscape of changing climate. Microorganisms.

[10] Sadanandan S, Ramkumar K, Pillai NP, Anuvinda P, Devika V, Ramanunni K (2022). Biorecognition elements appended gold nanoparticle biosensors for the detection of food-borne pathogens-A review. Food Control.

[11] Wang W, Wang L, Su J, Xu Z (2020). Antibiotic susceptibility, biofilm-forming ability, and incidence of class 1 integron of Salmonella spp Escherichia coli, and Staphylococcus aureus isolated from various foods in a school canteen in China. Foodborne Pathog Dis.

[12] Possas A, Pérez-Rodríguez F (2022). New insights into Cross-contamination of Fresh-Produce. Curr Opin Food Sci.

[13] Parrilli E, Tutino ML, Marino G (2022). Biofilm as an adaptation strategy to extreme conditions. Rendiconti Lincei. Scienze Fisiche e Naturali..

[14] Hayta EN, Ertelt MJ, Kretschmer M, Leileg O (2021). Bacterial materials: Applications of natural and modified biofilms. Adv Mater Interfaces.

[15] Dincer S, Uslu FM, Delik A (2020). Antibiotic resistance in biofilm. Bact Biofilms IntechOpen.

[16] Carrascosa C, Raheem D, Ramos F, Saraiva A, Raposo A (2021). Microbial biofilms in the food industry—A comprehensive review. Int J Environ Res Public Health.

[17] Mahendran R, Ramanan KR, Barba FJ, Lorenzo JM, López-Fernández O, Munekata PE (2019). Recent advances in the application of pulsed light processing for improving food safety and increasing shelf life. Trends Food Sci Technol.

[18] Cacciatore FA, Brandelli A, Malheiros PD (2021). Combining natural antimicrobials and nanotechnology for disinfecting food surfaces and control microbial biofilm formation. Crit Rev Food Sci Nutr.

[19] Wu B, Miraghaee S, Handa S, Gallou F (2022). Nanoparticles for catalysis in aqueous media. Curr Opin Green Sustainable Chem.

[20] Sana SS, Li H, Zhang Z, Sharma M, Usmani Z, Hou T (2021). Recent advances in essential oils-based metal nanoparticles: A review on recent developments and biopharmaceutical applications. J Mol Liq.

[21] Sultan M, Abdelhakim AA, Nassar M, Hassan YR (2022). Active packaging of chitosan film modified with basil oil encapsulated in silica nanoparticles as an alternate for plastic packaging materials. Food Biosci.

[22] Tian B, Liu Y (2021). Antibacterial applications and safety issues of silica-based materials: A review. Int J Appl Ceram Technol.

[23] Huang Y, Li P, Zhao R, Zhao L, Liu J, Peng S (2022). Silica nanoparticles: Biomedical applications and toxicity. Biomed Pharmacother.

[24] Iraji S, Ganji F, Rashidi L (2018). Surface modified mesoporous silica nanoparticles as sustained-release gallic acid nano-carriers. J Drug Delivery Sci Technol.

[25] Qi M, Chi M, Sun X, Xie X, Weir MD, Oates TW (2019). Novel nanomaterial-based antibacterial photodynamic therapies to combat oral bacterial biofilms and infectious diseases. Int J Nanomedicine.

[26] Tortuel D, Tahrioui A, Rodrigues S, Cambronel M, Boukerb AM (2020). Activation of the cell wall stress response in Pseudomonas aeruginosa infected by a Pf4 phage variant. Microorganisms.

[27] Zulfiqar U, Subhani T, Wilayat Husain S (2016). Synthesis of silica nanoparticles from sodium silicate under alkaline conditions. J Sol-Gel Sci Technol.

[28] Parvekar P, Palaskar J, Metgud S, Maria R, Dutta S (2020). The minimum inhibitory concentration (MIC) and minimum bactericidal concentration (MBC) of silver nanoparticles against Staphylococcus aureus. Biomater Invest Dent.

[29] Ahmed F, Mirani ZA, Ahmed A, Urooj S, Khan FZ, Siddiqi A (2022). Nanotubes formation in P. aeruginosa. Cells.

[30] Luo A, Wang F, Sun D, Liu X, Xin B (2022). Formation, development, and cross-species interactions in biofilms. Front Microbiol.

[31] Trubenová B, Roizman D, Moter A, Rolff J, Regoes RR (2022). Population genetics, biofilm recalcitrance, and antibiotic resistance evolution. Trends Microbiol.

[32] Flemming HC, van Hullebusch ED, Neu TR, Nielsen PH, Seviour T, Stoodley P (2023). The biofilm matrix: Multitasking in a shared space. Nat Rev Microbiol.

[33] Rather MA, Gupta K, Bardhan P, Borah M, Sarkar A, Eldiehy KS (2021). Microbial biofilm: A matter of grave concern for human health and food industry. J Basic Microbiol.

[34] Smirnov NA, Kudryashov SI, Nastulyavichus AA, Rudenko AA, Saraeva IN (2018). Antibacterial properties of silicon nanoparticles. Laser Phys Lett.

[35] Khan J, Tarar SM, Gul I, Nawaz U, Arshad M (2021). Challenges of antibiotic resistance biofilms and potential combating strategies: A review. 3. Biotech.

[36] Mirani ZA, Urooj S, Khan MN, Khan AB, Shaikh IA, Siddiqui A (2020). An effective weapon against biofilm consortia and small colony variants of MRSA. Iran J Basic Med Sci.

[37] Ullah A, Yin X, Wang F, Xu B, Mirani ZA, Xu B (2021). Biosynthesis of selenium nanoparticles (via Bacillus subtilis bsn313), and their isolation, characterization, and bioactivities. Molecules.

[38] Tian B, Liu Y, Chen D (2021). Adhesion behavior of silica nanoparticles with bacteria: Spectroscopy measurements based on kinetics, and molecular docking. J Mol Liq.

[39] Mirani ZA, Khan MN, Siddiqui A, Khan F, Aziz M, Naz S (2018). Ascorbic acid augments colony spreading by reducing biofilm formation of methicillin-resistant Staphylococcus aureus. Iran J Basic Med Sci.

[40] Mirani ZA, Naz S, Khan F, Aziz M, Khan MN, Khan SI (2017). Antibacterial fatty acids destabilize hydrophobic and multicellular aggregates of biofilm in Staphylococcus aureus. Antibiotics.

[41] Witten J, Ribbeck K (2017). The particle in the spider’s web: transport through biological hydrogels. Nanoscale.

[42] Pratten J, Barnett P, Wilson M (1998). Composition and susceptibility to chlorhexidine of multispecies biofilms of oral bacteria. Appl Environ Microbiol.

[43] Adeyeye SA, Ashaolu TJ (2021). Applications of nano-materials in food packaging: A review. J Food Process Eng.

[44] Winkler HC, Suter M, Naegeli H (2016). Critical review of the safety assessment of nano-structured silica additives in food. J Nanobiotechnology.

[45] Akhidime ID, Saubade F, Benson PS, Butler JA, Olivier S, Kelly P (2019). The antimicrobial effect of metal substrates on food pathogens. Food Bioprod Process.

